# Latent Class Analysis of Victimization Patterns and Associated Protective Factors among LGBTQ Youth

**DOI:** 10.3390/ijerph19169953

**Published:** 2022-08-12

**Authors:** Alberto Valido, Matthew Rivas-Koehl, Dane Rivas-Koehl, Dorothy L. Espelage, Timothy I. Lawrence, Luz E. Robinson

**Affiliations:** 1 School of Education, University of North Carolina at Chapel Hill, Chapel Hill, NC 27514, USA; 2 Department of Human Development and Family Studies, University of Illinois Urbana-Champaign, Urbana, IL 61801, USA; 3 College of Arts and Sciences Psychology Department, Prairie View A&M University, Prairie View, TX 77446, USA

**Keywords:** latent class analysis, sexual harassment, homophobic bullying, bullying victimization, protective factors

## Abstract

Youth victimization in schools remains a fervent public health issue, despite increased awareness of this issue, and this is especially true for marginalized populations like lesbian, gay, bisexual, transgender, and queer (LGBTQ) youth. Youth violence has been studied widely, but less research has sought to understand factors protective of violence victimization, particularly protective factors shared across multiple forms of violence. In the current study, we utilized latent class analysis to test patterns of three types of victimization: peer victimization (PV), homophobic name-calling victimization (HNCV), and sexual harassment victimization (SHV). In addition, we tested protective factors associated with experiencing these types of violence. Our sample included 4778 9–11th graders in the United States, of which about 15% identified as LGBTQ. Three unique classes of victimization emerged, suggesting that concurrent forms of violence occur among some groups of adolescents. LGBTQ youth were more likely to be members of classes which demonstrated higher levels of victimization. Consistent with previous literature, medical access, counseling access, family support, peer support, and spirituality emerged as significant protective factors associated with a lower risk of victimization. We discuss the implications of our findings with specific attention to protecting the wellbeing of SGM youth.

## 1. Introduction

Despite increasing acceptance and support for lesbian, gay, bisexual, transgender, and queer (LGBTQ) youth, students who identify as LGBTQ continue to experience disproportionately higher rates of interpersonal victimization at school compared to their heterosexual peers. For example, recent nationally representative data illustrated that more than half of LGBTQ youth have reported feeling unsafe at school because of their sexual identity, and almost all students have reported hearing anti-LGBTQ remarks in the past year [1]. Additionally, more than half of LGBTQ youth have reported experiencing verbal harassment and about one-quarter have experienced physical harassment based on their gender and sexual identities [1]. Such victimization rates have severe consequences on the mental health of these youth [2]. The Trevor Project’s most recent nationally representative survey of LGBTQ youth and mental health indicated that 45% of LGBTQ youth seriously considered, and 14% of these youth attempted, suicide in the past year [3]. Importantly, however, these disproportionate rates of victimization and subsequent reports of poor mental health are not inherently a result of one’s LGBTQ identity. Instead, heterosexist climates in which violence toward LGBTQ youth is allowed to continue must be implicated in this pattern [4,5]. As such, research must focus on understanding what factors may be protective of victimization for LGBTQ youth to close the gap in mental health disparities.

While much of the research has examined types of victimization in isolation, the current study sought to better understand patterns of victimization across multiple outcomes. In particular, we examined sexual harassment victimization (SHV), homophobic name-calling victimization (HNCV), and peer victimization (PV) using a latent class analysis (LCA) approach. We examined how LGBTQ identities were related to latent class membership and what factors were protective of victimization patterns to provide directions for improving the lives of LGBTQ youth.

### 1.1. Homophobic Name-Calling Victimization

The current study examines three related but distinct types of victimization that youth experience in schools. First, HNCV is a form of verbal harassment rooted in discrimination toward LGBTQ individuals and prejudice toward those who do not conform to normative standards of gender expression [6,7]. Thus, HNCV accounts for derogatory epithets often directed toward LGBTQ individuals, but not exclusively, as heterosexual youth also report experiencing HNCV [8,9]. Particularly for boys, using homophobic epithets may be a means of asserting one’s masculinity among peers by calling out others for not conforming to hegemonic standards of masculinity [10]. This is problematic as multiple studies have demonstrated the cyclical nature of this type of victimization, wherein those who experience HNCV are more likely to perpetrate this behavior [10,11], possibly as a means of renegotiating or reasserting their masculinity in a social hierarchy among their peers. Across gender and sexual identities, HNCV has been associated with adverse outcomes for overall wellbeing, including psychological distress, higher rates of substance use and depressive symptoms, and lowered self-esteem [8,12]. Protective factors of HNCV include increased social-emotional competencies, family and peer support, and positive school climates for LGBTQ students [9,13]. However, much of the existing research focuses on risk factors for experiencing victimization, so further studies are needed to confirm the most effective ways to intervene and prevent this type of victimization.

### 1.2. Peer Victimization

PV, or bullying, has been studied widely in recent years. General bullying or peer victimization often includes verbal and/or physical aggression directed at an individual perceived as less powerful by the perpetrator [14]. The harmful effects of bullying on youth outcomes are widespread, including negative psychosocial, physical, and academic consequences. For example, poorer mental health, increased likelihood of developing an illness, lower grades, and truancy have all been associated with being victimized by one’s peers [15,16,17]. Bullying affects students across all gender and sexual identities but is particularly prevalent for marginalized students such as those who identify as LGBTQ; nationally representative survey data show that an overwhelming majority (86.3%) of LGBTQ students experienced some form of harassment at school in the last year because of their sexual or gender identity [1]. Given that peer victimization encompasses general bullying behaviors that may escalate into more targeted or specific forms of victimization, it is likely that PV may be experienced alongside other forms of victimization like SHV and HNCV.

### 1.3. Sexual Harassment Victimization

In the United States, sexual harassment is legally considered by the Department of Education Office of Civil rights to be “unwelcome conduct of a sexual nature”, which can include “unwelcome sexual advances, requests for sexual favors, and other verbal, nonverbal, or physical conduct of a sexual nature” [18]. For youth, SHV often takes the form of sexual teasing and spreading rumors and is most frequently perpetrated by boys toward girls [19]. However, while SHV is often considered within heterosexual contexts (e.g., boys catcalling girls), LGBTQ youth experience even higher rates of SHV [1,2], perhaps because their gender and sexual identities may be already in question or paid particular attention to among their peers [20,21]. SHV is harmful to all youth, but girls and LGBTQ individuals appear to be at exceptionally high risk for reported outcomes of SHV, including poorer mental health and school performance [1,2,19,22]. Because of the nature of SHV, it has been linked to other forms of aggression, such as HNCV and PV [23]. In fact, similar risk and protective factors have been observed across these multiple domains of victimization [19,22,23,24].

### 1.4. Individual, Family, School, and Community Level Protective Factors

Factors protective of multiple forms of victimization for all youth are of utmost importance for scholars and practitioners to recognize, particularly for LGBTQ youth who experience an increased risk of victimization because of the stigma and discrimination they face in society. Further, because the types of victimization we examine in this study are related [23], it is plausible that there may be shared protective factors that practitioners and scholars could capitalize on to prevent concurrent violence and victimization. The social-ecological theory provides a useful framework for considering tiered levels of prevention and intervention across the multiple domains of youth’s lives. 

The social-ecological theory posits that individuals are simultaneously influenced by individual, family, community, and societal levels [25]. Protective factors of victimization in schools have been described at multiple levels of the social ecology. For example, at the individual level, spirituality has been shown to be protective of adverse outcomes [26,27]. Previous research has shown family and school-level factors like support from trusted adults, peer groups, and family members to be protective factors for youth across sexual and gender identities [9,13,26,27]. At the community level, we included access to medical and counseling services to explore whether these types of support may be protective against multiple forms of victimization, both of which have been supported by prior research [9]. All of these protective factors share a common thread of having access to support and resources. Still, there are unique implications based on the level of the social ecology in which the protective factor is situated. As such, we must measure protective factors across multiple levels to comprehensively support adolescent lives.

In the current study, we extend this previous research by examining these common protective factors in terms of latent class membership to understand which specific protective factors can be capitalized upon to prevent concurrent forms of victimization. At the broadest level, we also recognize that societal protective factors of LGBTQ victimization include combatting heteronormative, homophobic, and transphobic social climates. While extremely important to consider, the ideologies are not measured in the current study and are thus beyond the scope of this project.

### 1.5. Minority Stress Theory

Meyer’s (2003) [28] minority stress theory (MST) has been foundational in research exploring the experiences of LGBTQ populations. MST helps explain the excess stress LGBTQ individuals experience in a society that marginalizes non-heterosexual and cisgender identities. Due to this undue stress, MST posits that LGBTQ youth are at higher risk of adverse mental health outcomes. These experiences of stress exist on a spectrum of distal to proximal stressors, which differentially impact one’s wellbeing [28]. Distal stressors are defined as external impacts on marginalized individuals (e.g., discrimination and harassment), and proximal stressors are the internalization of adverse events and attitudes (e.g., internalized homophobia). These different stressors have a direct impact on the mental health outcomes of LGBTQ populations but have been seen to be mitigated by the community and social resources (e.g., affirming environments, connectedness to their community, etc.). In the current study, MST is applied to understand that the increased likelihood of LGBTQ youth experiencing multiple forms of victimization is underscored by their minoritized position in society. To interrupt the stress process that denigrates the health and wellbeing of LGBTQ youth, we must consider protective factors such as those highlighted by MST and the social-ecological theory.

### 1.6. Current Study

In the current study, we drew from a large and diverse sample of adolescents to examine patterns of victimization using LCA. In particular, this study aims to highlight the experiences of LGBTQ youth, given that a dearth of literature has sought to understand experiences specific to LGBTQ youth. Further, protective factors of adolescent victimization, particularly for LGBTQ youth, have not been examined to the extent that risk factors have; thus, the current research fills an important gap in the literature by investigating protective factors of victimization. Specifically, the present study examines six protective factors shown to be effective among adolescents: (1) medical access, (2) counseling access, (3) trusted adults, (4) family support, (5) peer support, and (6) spirituality, to better understand how to prevent multiple forms of adolescent violence victimization.

## 2. Materials and Methods

### 2.1. Participants

Participants (N = 4778) included students who participated in the baseline evaluation of a randomized clinical trial of the suicide prevention program Sources of Strength [29,30]. Twenty schools were recruited by contacting school districts in diverse areas across a western U.S. state. All 9–11th grade students in each school were invited to participate. The present study used data from all 20 schools collected during Fall 2017 (81% response rate). At the school level, the percentage of students receiving free and reduced-price lunch ranged from 1% to 88% (mean = 51%), with two schools having no free and reduced-price lunch programs. Eleven schools were in urban counties, eight were in rural counties, and one was in a frontier county. See Table 1 for sample demographics and descriptive statistics on study variables.

### 2.2. Procedure

The study was approved by four institutional review boards, which authorized a passive waiver of parental consent for this study. Study information was provided to eligible students, and those who provided assent were invited to complete an online survey. Data were collected in each classroom during school hours under the supervision of researchers. Most students completed the online survey in English, though translated surveys were used for Spanish-speaking students, and the survey was also offered in braille in one school. Students were given resources after survey completion related to suicidal concerns, depression, and sexual violence.

### 2.3. Measures

*Demographics.* Each student was asked to report sex, whether or not they identified as transgender, age, sexual orientation, and race/ethnicity. For race/ethnicity, sex, and sexual orientation, students were asked to check all options that applied.

*Protective Factors.* Six single-item indicators were developed for this study to assess protective factors across multiple domains. Students were asked, “How much, if at all, in the last six months would you agree or disagree with the following statements about yourself?”. The six protective factors measured are as follows: (1) Medical access: “I get any medical services I need”; (2) Counseling access: “If needed, I could get counseling or help”; (3) Trusted adults: “I have friendships with adults that I trust”; (4) Family support: “I feel supported and cared for by my family”; (5) Peer support: “I have positive, caring friends”; and (6) Spirituality: “I feel very spiritual in my faith, beliefs, and culture”. Response options for all items include 0 (Strongly Disagree), 1 (Disagree), 2 (Agree), 3 (Strongly Agree). For the present study, all items were binarized with “0” indicating disagreeing or strongly disagreeing to the protective factor and “1” indicating agreeing or strongly agreeing to the item.

*Sexual Harassment Victimization (SHV).* SHV was measured using four items adapted from the modified version of the American Association of University Women Sexual Harassment Survey—Victimization Scale [31,32]. Students are asked, “How often, if at all, in the past six months have others done the following things to you at school when you did not want them to?” The four items include: (1) “made sexual comments, jokes, gestures, or looks”; (2) “showed, gave, or left you sexual pictures, photographs, illustrations, messages, or notes”; (3) “wrote sexual messages/graffiti about you on bathroom walls, in locker rooms, etc.”; (4) “spread sexual rumors about you”. Response options include 0 (Never), 1 (1–2 times), 2 (3–4 times), 3 (5–6 times), and 4 (7 or more times) on a five-point Likert-type scale. For this study, each item in the scale was binarized with “0” indicating never experiencing SHV and “1” indicating any experience of victimization during the past six months.

*Homophobic Name-Calling Victimization (HNCV).* Homophobic name-calling victimization was measured using the five-item Homophobic Content Target Scale [33,34]. To assess victimization, students are asked, “How many times in the last 30 days did the following individuals say homo, gay, lesbo, or fag to you?” Students were then presented with five items: (1) a friend, (2) someone who does not know you well, (3) someone that does not like you, (4) someone who thought you were gay or lesbian, and (5) someone who did not think you were gay or lesbian. Response options include 0 (Never), 1 (1–2 times), 2 (3–4 times), 3 (5–6 times), and 4 (7 or more times) on a five-point Likert-type scale. Previous exploratory and confirmatory factor analyses supported the factor structure of this measure [33]. Cronbach’s alpha coefficient was 0.80 for this study. For this study, each item in the scale was binarized with “0” indicating never experiencing HNCV and “1” indicating any experience of victimization.

*Peer Victimization (PV).* The four-item University of Illinois Victimization Scale [35] assessed victimization from peers. Students are asked how often the following have happened to them in the past 30 days: (1) “Other students called me names”; (2) “Other students made fun of me”; (3) “Other students picked on me”; and (4) “I got hit and pushed by other students”. Response options include 0 (Never), 1 (1–2 times), 2 (3–4 times), 3 (5–6 times), and 4 (7 or more times) on a five-point Likert-type scale. Exploratory and confirmatory factor analyses have supported the construct validity of this scale, and scores have converged with peer nominations of victimization [35]. For this study, each item on the scale was binarized with “0” indicating never experiencing PV and “1” indicating any experience of victimization.

### 2.4. Analysis Plan

We conducted a Latent Class Analysis (LCA) to estimate the associations between protective factors and the likelihood of membership in heterogeneous SHV, HNCV, and PV classes. LCA is a person-centered approach where individuals are assigned to classes or subgroups by using indicator variables to model the hidden heterogeneity in the sample [36]. We employed a “classify then analyze” approach where: (1) latent classes are first determined using observed indicators; (2) participants are given a probability of belonging to each of the classes and are assigned to the class with the highest posterior probability; and (3) a model is run to predict the final latent class membership while controlling for the error or uncertainty associated with the class classification [37]. The LCA was conducted using the manual three-step approach (auxiliary function 3RSTEP in Mplus 8.4), which automatically implements the classify then analyze approach to avoid the influence of predictor variables in determining the class enumeration [36,37,38].

In the first step, we conducted a series of six LCA models with an increasing number of classes. We then compared each model using several model fit indices and extant theory to determine the appropriate number of classes. The six-class model failed to converge and was not included in these analyses. To estimate model fit, we used the following indices: −2 Log Likelihood (−2LL), Akaike Information Criteria (AIC), Bayesian Information Criteria (BIC), Sample-Size Adjusted BIC (Adj. BIC), Consistent Akaike Information Criteria (CAIC), Approximate Weight of Evidence Criterion (AWE), the bootstrapped likelihood ratio test (BLRT), and the Lo–Mendell–Rubin adjusted likelihood ratio test (LMRT) [39,40,41]. In addition, we examined entropy, the percentage of participants in the smallest class, and the number of parameters estimated to decide the most parsimonious number of classes.

Additionally, we examined the percentages of maximum likelihood starting values where the best log-likelihood was successfully replicated. A lower percentage was considered an indication of a poorer model fit. Improved model fit was determined by decreasing values of −2LL, AIC, BIC, SSBIC, CAIC, and AWE when comparing a model with k classes and a model with k − 1 classes. The BLRT estimates the significance of the reduction in −2LL between the k and k − 1 models [42]. For entropy, values above 0.80 indicate adequate separation between classes [43].

In the second step, we estimated the most likely class for each individual using the latent class posterior probabilities obtained in the first step [36]. Lastly, we used multinomial logistic regression to estimate the associations between the likelihood of class membership, and each of the six included protective factors, as well as single-item indicators for each sexual orientation (gay/lesbian, bisexual, questioning, and other sexual orientation) compared to heterosexual, gender (female and other gender), and race/ethnicity (Black or African American, Hispanic, and Multiracial). The reference categories for the demographic variables included heterosexual for sexual orientation, male for gender, and White for race/ethnicity. Odds ratios for the multinomial logistic regression models indicate the association between each predictor and the likelihood of belonging to each class compared to a reference class.

Lastly, in the third set of models, we tested whether each protective factor moderated the associations between LGBTQ identity and the probability of class membership. Unfortunately, the models failed to converge due to the high number of interactions between multiple LGBTQ identities and protective factors. Results from the interaction analysis are not presented here for simplicity.

Further, we computed descriptive statistics and bivariate correlations and examined the prevalence of each outcome according to students’ final latent class. Bivariate correlations were calculated at the item level to evaluate whether multicollinearity would influence the latent class classifications. Bivariate correlations at the item level did not show evidence of multicollinearity *(r* > 0.90), with the highest absolute value of the correlation coefficients being *r* = 0.73 (Table 2). Additionally, we examined bivariate correlations between class indicators within each class to evaluate the assumption of local independence. The highest absolute value of the within-class correlation coefficients was *r* = 0.63 suggesting there were no violations of the assumption of local independence.

Full Information Maximum Likelihood (FIML) was used to handle missing data which ranged from 1.2% to 3.4%. We used the Chi-Square test for Missing Completely at Random (MCAR) in Mplus to determine whether missingness due to variables not included in the model impacted model estimates. The MCAR test was not significant, suggesting that the data were missing completely at random; thus, FIML was an appropriate strategy to handle missing data. Further, all observations were used in the analysis without excluding individual cases with unusual patterns of responses across outcomes (e.g., multivariate outliers). This allowed us to utilize all available information to inform the class model selection.

## 3. Results

### 3.1. Descriptive Statistics

Rates of victimization (Table 1) were high across all participants: 35.9% reported experiencing PV, 49.3% reported HNCV, and 29.6% reported experiencing SHV. The prevalence of each protective factor was relatively high, ranging from 75.9% to 81.8%.

The percentage of LGBTQ students in the sample was also high (15.1%); about 7.1% identified as bisexual, 2% as gay/lesbian, 2.6% as questioning, 3.4% as other sexual orientation, and 1.1% identified as transgender. Table 2 presents bivariate correlations between all study variables.

### 3.2. Latent Class Results

Table 3 shows model fit statistics for models with an increasing number of classes. Decreasing values for the −2LL, AIC, BIC, Adj. BIC, CAIC, and AWE supported increasing the number of classes to a five-class solution. The five-class model was also supported by statistically significant LMRT and BLRT (*p* < 0.001). However, a closer examination of the models revealed issues with the percentage of starting values converging (37%) in the five-class solution, as well as a relatively low percentage of participants in the smallest class. The four-class solution had a higher percentage of starting values converging (72%), but the smallest class still had a similarly low number of participants (9%) as the five-class solution.

**Figure 1:**
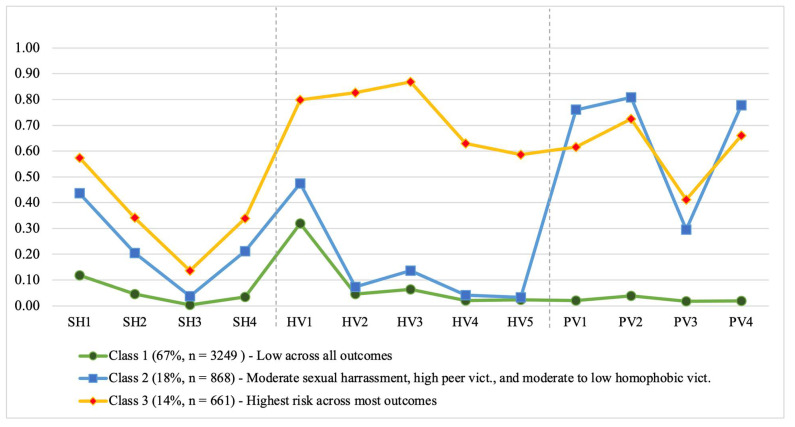
Graphical Representation of Endorsement Probabilities for each item by Latent Class (N = 4778). Note: Sexual Harassment: SHV1 = Made sexual comments, jokes, gestures, or looks; SHV2 = showed, gave, or left you sexual pictures, photographs, illustrations, messages, or notes; SHV3 = wrote sexual messages/graffiti about you on bathroom walls, in locker rooms, etc.; SHV4 = spread sexual rumors about you; Homophobic Victimization: (How many times in the last 30 days did the following individuals say homo, gay, lesbo, or fag to you?): HV1 = a friend?; HV2 = someone who does not know you well?; HV3 = someone who does not like you?; HV4 = someone who thought I was gay or lesbian?; HV5 = someone who did not think I was gay or lesbian?; Peer Victimization: PV1 = other students picked on me; PV2 = other students called me names; PV3 = I got hit or pushed by others; PV4 = other students made fun of me.

An examination of the entropy value (0.89) suggested that the three-class model offered the best fit and the highest degree of separation between the classes. The three-class solution also had the highest percentage of converging starting values (92%) compared to the four-class and five-class solutions. The three-class solution was also more parsimonious in the number of parameters estimated compared to the four and five-class models and had a relatively larger number of participants in the smallest class (14%). Lastly, an examination of the endorsement probabilities for each class in the three-class solution (Table 4 & Figure 1) offered the highest interpretability and alignment with theory. The three-class solution was then selected as the best fitting model and used in all subsequent analyses.

Table 4 and Figure 1 show the endorsement probabilities for each item in the three latent classes. About 67% of the sample was in Class 1, which was characterized by low endorsement across most of the items and the lowest level of victimization compared to the other classes. Class 2 included about 18% of the sample and was moderately high in SHV, high in PV, and moderate to low in HNC. Lastly, Class 3 had 14% of the sample and showed the highest levels of victimization across most of the outcomes with high SHV, HNC, and PV.

Table 5 shows the number and percentage of participants endorsing each item in the three latent classes. Consistent with item probabilities, results showed that Class 3 and Class 2 had higher percentages of participants reporting victimization experiences compared to Class 1.

Table 6 presents the percentage of participants classified in each class according to gender, sexual orientation, race/ethnicity, and prevalence of each protective factor. Class 1 (low risk) was characterized by a higher endorsement of each protective factor compared to Class 2 and Class 3. According to gender, Class 3 (highest risk across all outcomes) had a higher proportion of male students while Class 2 (moderate risk) had a higher proportion of female students.

Furthermore, Class 3 had a significantly higher proportion of students who identify as other gender, transgender, gay or lesbian, bisexual, and students who identify as other sexual orientation. According to race/ethnicity, a higher proportion of Multiracial students were classified in Class 3, while a greater proportion of Hispanic students were classified in Class 1. Lastly, a higher proportion of White students were classified in Class 2 and Class 3 compared to Class 1. All statistical comparisons were significant at *p* < 0.05.

### 3.3. Multinomial Logistic Regression Results

According to its low probability of victimization, Class 1 was selected as the reference class to indicate the probability of belonging to a moderate or high class of victimization compared to low or no victimization. The odds ratios in Table 7 indicate the association between each of the predictors and the odds of experiencing moderate (Class 2) to high (Class 3) risk of SHV, HNC, and PV, compared to the odds of experiencing the lowest level of risk (Class 1).

### 3.4. Protective Factors

Several protective factors were associated with lower odds of belonging to a high-risk class compared to a low-risk class (Table 7). Participants who reported having medical access (O.R. = 0.67, *p* < 0.001) and counseling access (O.R. = 0.72, *p* < 0.001) were associated with a decrease in the odds of being classified in Class 2 compared to Class 1. In other words, medical and counseling access were associated with a 33% and 28% lower probability of being in Class 2 than Class 1.

Furthermore, participants who reported counseling access were 30% less likely to be classified in Class 3 (O.R. = 0.70, *p* < 0.001) than Class 1. The protective factors family support (O.R. = 0.64, *p* < 0.001) and peer support (O.R. = 0.54, *p* < 0.001) were also associated with lower odds of belonging to the high-risk Class 3 compared to Class 1. Students who reported feeling supported by their families were 36% less likely to be classified in Class 3, while those who reported having peer support were 46% less likely. Lastly, spirituality was associated with lower odds of being in Class 2 (O.R. = 0.73, *p* < 0.001) and Class 3 (O.R. = 0.66, *p* < 0.001), compared to Class 1. In other words, students who reported being spiritual were 27% and 34% less likely to be in Class 2 and Class 3, respectively.

### 3.5. Sexual Orientation

Regarding sexual orientation, when compared to heterosexual students, bisexual (O.R. = 6.01, *p* < 0.001), gay and lesbian (O.R. = 5.37, *p* < 0.001), and youth who reported other sexual orientation (O.R. = 3.59, *p* < 0.001) were associated with the highest odds of being classified in Class 3 across all predictors. Bisexual students had the highest risk and were six times more likely to be classified in Class 3 compared to Class 1; gay and lesbian youth were about five times more likely; and adolescents who identified as other sexual orientation were about 3.5 times more likely to be in the highest risk category. Conversely, identifying as questioning (O.R. = 0.59, *p* < 0.05) was significantly associated with 41% lower odds of belonging to Class 2 than Class 1.

### 3.6. Other Demographic Characteristics

Additionally, there were significant associations between age in years and the odds of being classified in Class 2 (O.R. = 0.88, *p* < 0.001) or Class 3 (O.R. = 0.83, *p* < 0.001). An added year of age was associated with 12% and 17% lower odds of being in Class 2 and Class 3, respectively. Identifying as female was also associated with 36% higher odds (O.R. = 1.36, *p* < 0.01) of being classified in the moderate-risk Class 2 and 64% lower odds of being classified in the high-risk Class 3 (O.R. = 0.46, *p* < 0.001) compared to the low-risk Class 1. Lastly, compared to students who identified as White, Hispanic students had 29% lower odds of being classified in Class 2 (O.R. = 0.71, *p* < 0.001) and 35% lower odds of being classified in Class 3 (O.R. = 0.65, *p* < 0.001).

## 4. Discussion

The present study used LCA to explore latent classes of SHV, HNCV, and PV in a diverse sample of high school students. In addition, we examined the associations between protective factors, sexual orientation, gender, and race/ethnicity on the likelihood of class membership. In particular, we center our discussion on the class membership patterns of LGBTQ youth, as this population is understudied in terms of protective factors of victimization despite generally experiencing higher rates of victimization than heterosexual youth.

Findings showed that a three-class solution better fit the data than a four-class or five-class solution. Class 1 (67%) was characterized by low levels of risk across all outcomes and contained the majority of the sample. Class 2 (18%) showed moderate levels of risk for SHV, high for PV, and medium to low for HNCV. Lastly, Class 3 (14%) was characterized by high risk across all outcomes. The distribution of students in Classes 2 and 3 suggests that an alarming 32% of students reported some experience of SHV, HNCV, and PV at school.

In agreement with our hypotheses, several protective factors were associated with a lower likelihood of belonging to the medium (Class 2) or high-risk class (Class 3) compared to the lowest risk class (Class 1). Consistent with previous literature, medical access, counseling access, family support, peer support, and spirituality emerged as significant protective factors associated with a lower risk of victimization [9,13,26]. Additionally, we found significant associations between a student’s gender, sexual orientation, race/ethnicity, and the likelihood of belonging to a high-risk class. Specifically, students who identify as bisexual, gay/lesbian, and other sexual orientation (i.e., they identified themselves as not any of the listed options) were on average 3.5 to 6 times more likely to be in the highest risk class. Female students were more likely to be in Class 2, which experienced moderate levels of risk, and Hispanic students were less likely to be in a high-risk class. Our findings contribute to the previous literature by simultaneously examining multiple forms of victimization, examining how diverse identities relate to class membership, and providing directions for future prevention research among adolescents.

Medical and counseling access were protective factors significantly associated with a lower likelihood of belonging to the class with moderate victimization experiences (Class 2), and counseling access was related to a lower likelihood of belonging to the class with high victimization across all outcomes (Class 3). These findings support previous findings that have shown associations between counseling and medical access as a shared protective factor for multiple forms of violence, particularly among LGBTQ youth [9,26,44,45]. Counseling and medical access could be protective by offering youth mental and physical health resources to cope with experiences of violence at school. Counselors and health care professionals can also be resources for youth to seek help when victimized. These professionals can help escalate students’ concerns to school officials, parents, and law enforcement. These findings support increasing mental health and medical access at school, as they may be effective against multiple forms of victimization.

Furthermore, the present study supports previous research that identified family support as a strong predictor of positive adolescent outcomes [46,47,48,49,50]. Supportive families with warm and affectionate relationships, good communication, and high parental involvement may protect against multiple forms of victimization by fostering a positive home environment, identity development, and increasing adolescent coping strategies [51]. Poteat and colleagues (2011) [52] found that, in a large sample of middle and high school students (N = 15,293), high parental support was associated with lower HNCV for white and racial minority youth. In addition, a meta-analysis by Lereya and colleagues (2013) [51] found that high parental involvement and supervision were associated with lower rates of bullying victimization. Furthermore, family warmth and connection have been shown to promote resilience and positive adjustment among bully victims [53]. Conversely, low family support and family rejection have been associated with higher rates of suicide, sexual risk-taking, homelessness, and substance abuse [54,55,56]. Although little is known about how family support is associated with SHV, our findings also align with studies showing protective associations between family support and the incidence of SHV [57,58]. These findings emphasize the importance of family support among adolescents and highlight the need for future research examining the role of the family in preventing multiple forms of victimization at school.

Similarly, we found a significant association between peer support and a lower likelihood of belonging to the high-risk class of SHV, HNCV, and PV compared to the low-risk class. These findings are consistent with research showing that supportive friendships can be protective against experiences of victimization at school [59,60,61]. Furthermore, a literature review of protective factors associated with homophobic bullying found that higher peer support was associated with lower HNCV [13]. The role of peers can explain these findings as socializing agents contributing to the development of social-behavioral norms, including the endorsement of traditional masculinity attitudes and gender norms, heterosexism, cissexism, and homophobia that may contribute to high rates of victimization [11,13,34,62]. In turn, supportive and caring friends can create a positive group climate that discourages victimization. For instance, Poteat (2008) [63] found that lower homophobic attitudes within one’s peer group were associated with lower HNCV, and Poteat and colleagues (2009) [64] showed that willingness to remain friends with gay/lesbian friends was associated with lower bullying and improved school climate. Adolescents may also turn to supportive friends to prevent victimization and find help and resources to cope with its consequences.

Having the support of their families and friends is especially important for LGBTQ adolescents as they undergo a critical period of sexual and identity development and may experience rejection from their peers and families [65]. Although we could not examine whether the protective factors directly moderated the associations between LGBTQ identity and latent class membership, we found that family and peer support were associated with a lower likelihood of belonging to a high-risk class with a higher number of LGBTQ youth. Similarly, spirituality emerged as a significant protective factor of belonging to a high risk-class. Previous research has shown spirituality to be protective of adverse outcomes for GSM youth [26,27]. Scholars have demonstrated that LGBTQ individuals who are spiritually involved may experience many positive benefits to their wellbeing, including greater acceptance of their own LGBTQ identity and more positive relationships with others [66]. Such factors may help explain why spirituality emerged as a protective factor in our study.

While we have discussed patterns that can be seen in our sample, to further the knowledge of LGBTQ students’ experiences in schools, it is important to clarify that being a sexual or gender minority does not automatically assign a student to a higher risk category. A person with an LGBTQ identity is not inherently at risk for experiencing victimization; having an LGBTQ identity in a society that privileges heterosexuality, cisnormativity, and other hegemonic identities over ones that do not align with a cis- and hetero-normative culture creates an environment that positions LGBTQ individuals to be at a higher risk to experience undue stress. This is especially important to recognize in light of the numerous anti-LGBTQ bills that have been proposed and passed in many states. Considering the social climate, norms, and hegemonies that exist within an environment when exploring the lives of LGBTQ individuals is vital for understanding their experiences within the context and to avoid placing causality on having an LGBTQ identity. As the current study shows, contextual influences, such as support systems for LGBTQ individuals, can impact how various forms of stress and discrimination are experienced.

### 4.1. Limitations

While this study has significant strengths, including a large sample size, assessments of multiple forms of victimization, and exploring protective factors against victimization for LGBTQ youth, it is not without limitations. First, data were collected in one U.S. state, so the findings should be interpreted cautiously and generalized only to similar samples. Second, the dataset included few African American youth, so future studies should consider the impact of protective factors on the association between victimization experiences for African American LGBTQ youth. Similarly, we did not examine the effect of protective factors on victimization for youth with intersecting identities (e.g., race, gender, and sexual identity), which should be a priority for researchers who can secure large and diverse samples. Intersectional experiences need to be examined to understand the compounding sources of stress that multiple marginalized LGBTQ students face in climates that privilege dominant identities (e.g., whiteness, cisgender, heterosexuality). Third, we were unable to determine the impact of other contextual variables, such as socioeconomic status at the individual-level and neighborhood or school characteristics which were not collected in this survey. Fourth, the assessment of protective factors included single-item indicators, which are often associated with low reliability, and may have impacted the validity of the findings. However, although single items were utilized, the findings where protective factors significantly predicted less victimization suggest that these protective factors are quite robust. Lastly, between-group analyses inherently have a comparative component underlying them, and work with LGBTQ populations is compared to heterosexual populations. We understand that our analyses shared this underlying comparison and that within-group analyses for LGBTQ students would offer a closer look into the differences within the LGBTQ community and align with suggestions from other LGBTQ scholars [67]. Within-group analyses will also help to parse apart the within-group differences in LGBTQ students’ experiences in schools and at home to help avoid homogenizing LGBTQ populations.

### 4.2. Implications

This study highlights several protective factors of youth victimization for practitioners to consider in their work. Given that medical access, counseling access, family support, peer support, and spirituality were protective for sexual minority youth in this study, practitioners, researchers, and policymakers may invest in programs that target these resources in the social ecologies of sexual minority youth to prevent victimization. Because victimized youth often experience shame and guilt, which decreases their chances of disclosing information [68,69], therapists can include “safe” family members in session while utilizing trauma focus cognitive behavioral therapy (TF-CBT) to identify trauma symptoms and help youth develop adaptive coping mechanisms to reduce the severity of distressful and intrusive thoughts [70,71]. As prior literature suggests that therapists incorporating spirituality in sessions could serve as a mindfulness activity and increase psychological flexibility among sexually victimized youth [72,73], therapists should first understand the sense of spirituality of the youth and then, with permission, incorporate practices that align with their clients’ spiritual beliefs.

Sexual minority youth are not inherently at risk for experiencing various forms of victimization, rather, it is the conditions of their social ecologies that place them at risk. This underscores the continued need for schools and communities to address discriminatory climates, policies, and practices that enable high victimization rates to continue. For example, school-based violence prevention approaches will be enhanced if they are supported through anti-bullying legislation and policies that are comprehensive and enumerated [74,75,76]. Comprehensive and enumerated policies about bullying and discrimination are ones that explicitly state protection based on enumerated personal characteristics, including sexual orientation, gender identity/expression, and race/ethnicity.

As violence prevention research moves towards a strengths-based lens, the findings from this research emphasize the need to explore protective factors for victimization among this population and their intersecting identities (e.g., gender, race/ethnicity, age). Understanding how protective factors for violence and promotive factors for wellbeing present for sexual minority youth is imperative to violence prevention efforts in various settings (e.g., home, school, community, workplace).

## 5. Conclusions

The current study utilized a latent class analysis to determine what types of victimization were concurrently reported among youth during high school. In particular, we paid attention to class membership specific to LGBTQ youth and the protective factors that attenuated class membership based on victimization. Alarmingly, LGBTQ youth were shown to have an exceptionally high risk of belonging to a class with a higher risk of victimization. We also found promising results for violence prevention. Of the protective factors we examined, medical access, counseling access, family support, peer support, and spirituality were all associated with a lower risk of victimization, suggesting the importance for school and community practitioners to bolster these resources and forms of support for youth.

## Figures and Tables

**Table 1:** Descriptive statistics.

N = 4778	*n*	%
Age (Mean/SD)	14.97	0.92
Gender		
Male	2914	49.9%
Female	2297	47.5%
Transgender	55	1.1%
Other Gender	74	1.5%
Sexual Orientation		
Bisexual	338	7.1%
Gay/Lesbian	97	2.0%
Questioning	122	2.6%
Other Sexual Orientation	160	3.4%
Heterosexual	4046	84.9%
Race/Ethnicity		
Hispanic	2230	46.2%
White	1786	37.0%
Black or African American	83	1.7%
Asian	118	2.4%
American Indian or Alaskan Native	48	1.0%
Native Hawaiian or Pacific Islander	15	0.3%
Multiracial	550	11.4%
Victimization Outcomes ^a^		
Peer Victimization	1668	35.9%
Homophobic Victimization	2326	49.3%
Sexual Harassment Victimization	1378	29.6%
Protective Factors ^b^		
Medical Access	3579	81.1%
Counseling Access	3372	75.9%
Trusted Adults	3657	81.8%
Family Support	3863	86.0%
Peer Support	4045	90.5%
Spirituality	3078	69.6%

^a^ Counts and percentages indicate experiencing victimization at least once. ^b^ Counts and percentages indicate agreeing or strongly agreeing to the protective factor.

**Table 2:** Bivariate Correlations.

	1	2	3	4	5	6	7	8	9	10	11	12	13	14	15	16	17	18
1—Medical access	-																	
2—Counseling access	0.45	-																
3—Trusted Adults	0.34	0.39	-															
4—Family Support	0.34	0.34	0.40	-														
5—Friend Support	0.30	0.29	0.39	0.40	-													
6—Spirituality	0.29	0.32	0.35	0.32	0.29	-												
7—SVV1	−0.09	−0.11	−0.06	−0.12	−0.06	−0.12	-											
8—SVV2	−0.09	−0.12	−0.07	−0.12	−0.07	−0.11	0.44	-										
9—SVV3	−0.11	−0.10	−0.08	−0.10	−0.12	−0.06	0.22	0.38	-									
10—SVV4	−0.11	−0.10	−0.08	−0.16	−0.10	−0.10	0.41	0.40	0.32	-								
11—HV2	−0.06	−0.09	−0.09	−0.12	−0.09	−0.11	0.23	0.20	0.19	0.20	-							
12—HV3	−0.09	−0.07	−0.05	−0.10	−0.08	−0.12	0.23	0.20	0.19	0.23	0.59	-						
13—HV4	−0.06	−0.08	−0.07	−0.10	−0.09	−0.11	0.24	0.17	0.21	0.23	0.53	0.49	-					
14—HV5	−0.04	−0.07	−0.05	−0.08	−0.08	−0.10	0.21	0.20	0.21	0.18	0.49	0.46	0.50	-				
15—PV1	−0.11	−0.10	−0.10	−0.12	−0.11	−0.13	0.32	0.23	0.17	0.28	0.28	0.31	0.24	0.21	-			
16—PV2	−0.12	−0.13	−0.07	−0.12	−0.10	−0.12	0.34	0.25	0.17	0.27	0.32	0.35	0.28	0.26	0.67	-		
17—PV3	−0.12	−0.13	−0.08	−0.10	−0.09	−0.09	0.23	0.23	0.22	0.20	0.29	0.28	0.24	0.25	0.43	0.45	-	
18—PV4	−0.12	−0.12	−0.10	−0.12	−0.12	−0.14	0.31	0.22	0.17	0.29	0.30	0.33	0.27	0.24	0.73	0.70	0.46	-

Note: Red = Strong negative association; Orange = Moderate negative association; Yellow = Moderate positive association; Green = Strong positive association; Sexual Harassment: SHV1 = Made sexual comments, jokes, gestures, or looks; SHV2 = showed, gave, or left you sexual pictures, photographs, illustrations, messages, or notes; SHV3 = wrote sexual messages/graffiti about you on bathroom walls, in locker rooms, etc.; SHV4 = spread sexual rumors about you; Homophobic Victimization (How many times in the last 30 days did the following individuals say homo, gay, lesbo, or fag to you?): HV1 = a friend?; HV2 = someone who does not know you well?; HV3 = someone who does not like you?; HV4 = someone who thought I was gay or lesbian?; HV5 = someone who did not think I was gay or lesbian?; Peer Victimization: PV1 = other students picked on me; PV2 = other students called me names; PV3 = I got hit or pushed by others; PV4 = other students made fun of me.

**Table 3:** Latent Class Analysis (LCA) Model Fit Statistics.

Model	npar	Entropy	Converged (%) ^a^	Smallest Class (%)	−2LL	AIC	BIC	Adj. BIC	CAIC	AWE	LMRT *p*-Value	BLRT *p*-Value
1-class	13	n/a	100%	100%	53,489.11	53,515.11	53,599.24	53,557.93	53,549.94	53,623.77	-	-
2-class	27	0.88	100%	32%	43,869.69	43,923.69	44,098.43	44,012.63	43,996.03	44,149.37	*p* < 0.001	*p* < 0.001
**3-class**	**41**	**0.89**	**92%**	**14%**	**42,100.39**	**42,182.39**	**42,447.73**	**42,317.45**	**42,292.24**	**42,525.08**	***p* < 0.001**	***p* < 0.001**
4-class	55	0.85	72%	9%	40,954.01	41,064.01	41,419.96	41,245.19	41,211.37	41,523.73	*p* < 0.001	*p* < 0.001
5-class	69	0.86	37%	9%	40,460.33	40,598.33	41,044.88	40,825.62	40,783.20	41,175.06	*p* < 0.001	*p* < 0.001

Note: Bold indicates the best fitting class; ^a^ Percentage of starting values that converged; −2LL = −2 log likelihood; npar = number of parameters; AIC = Akaike information criterion; BIC = Bayesian information criterion; Adj. BIC = adjusted Bayesian information criterion; AWE = approximate weight of evidence criterion; LMRT = Vuong–Lo–Mendell–Rubin likelihood ratio test; BLRT = bootstrapped likelihood ratio.

**Table 4:** Endorsement Probability for Each Item by Latent Class.

Items		Class 1 ^a^ *n* = 3249		Class 2 ^b^ *n* = 868		Class 3 ^c^ *n* = 661	
		Prob.	S.E.	Prob.	S.E.	Prob.	S.E.
Sexual Harassment Victimization—How often, if at all, in the past six months have others done the following things to you at school when you did not want them to?							
SHV1.	Made sexual comments, jokes, gestures, or looks	0.12	0.01	0.44	0.02	0.57	0.02
SHV2.	Showed, gave, or left you sexual pictures, photographs, illustrations, messages, or notes	0.05	0.01	0.21	0.02	0.34	0.02
SHV3.	Wrote sexual messages/graffiti about you on bathroom walls, in locker rooms, etc.	0.00	0.00	0.04	0.01	0.14	0.02
SHV4.	Spread sexual rumors about you	0.03	0.00	0.21	0.02	0.34	0.02
Homophobic Victimization—How many times in the last 30 days did the following individuals say homo, gay, lesbo, or fag to you?							
HV1.	A friend?	0.32	0.01	0.48	0.02	0.80	0.02
HV2.	Someone who does not know you well?	0.05	0.00	0.07	0.01	0.83	0.03
HV3.	Someone who does not like you?	0.06	0.01	0.14	0.02	0.87	0.02
HV4.	Someone who thought I was gay or lesbian?	0.02	0.00	0.04	0.01	0.63	0.03
HV5.	Someone who did not think I was gay or lesbian?	0.02	0.00	0.03	0.01	0.59	0.03
Peer Victimization—How often the following happened at school in the last 30 days							
PV1.	Other students picked on me	0.02	0.00	0.76	0.03	0.62	0.03
PV2.	Other students called me names	0.04	0.01	0.81	0.02	0.73	0.02
PV3.	I got hit or pushed by others	0.02	0.00	0.30	0.02	0.41	0.02
PV4.	Other students made fun of me	0.02	0.00	0.78	0.03	0.66	0.03

Note: ^a^ Class 1 (67%)—Low across all outcomes, ^b^ Class 2 (18%)—Moderate sexual harassment, high peer victimization, and moderate to low homophobic victimization, ^c^ Class 3 (14%)—Highest risk across most outcomes, Prob. = probability, S.E.= standard error.

**Table 5:** Descriptive Statistics for Each Item by Latent Class.

		Class 1 ^a^ *n* = 3249	Class 2 ^b^ *n* = 868	Class 3 ^c^ *n* = 661	Sig. Comparisons ^d^
Sexual Harassment Victimization—How often, if at all, in the past six months have others done the following things to you at school when you did not want them to?					
SHV1.	Made sexual comments, jokes, gestures, or looks	381 (12%)	383 (45%)	368 (57%)	2 > 1; 3 > 1, 2
SHV2.	Showed, gave, or left you sexual pictures, photographs, illustrations, messages, or notes	138 (4.4%)	184 (22%)	222 (35%)	2 > 1; 3 > 1, 2
SHV3.	Wrote sexual messages/graffiti about you on bathroom walls, in locker rooms, etc.	6 (0.2%)	34 (4.0%)	89 (14%)	2 > 1; 3 > 1, 2
SHV4.	Spread sexual rumors about you	109 (3.4%)	181 (21%)	225 (35%)	2 > 1; 3 > 1, 2
Homophobic Victimization—How many times in the last 30 days did the following individuals say homo, gay, lesbo, or fag to you?					
HV1.	A friend?	1025 (32%)	414 (48%)	516 (80%)	2 > 1; 3 > 1, 2
HV2.	Someone who does not know you well?	150 (4.8%)	55 (6.5%)	539 (85%)	3 > 1, 2
HV3.	Someone who does not like you?	207 (6.6%)	109 (13%)	565 (89%)	2 > 1; 3 > 1, 2
HV4.	Someone who thought I was gay or lesbian?	68 (2.2%)	28 (3.3%)	414 (65%)	3 > 1, 2
HV5.	Someone who did not think I was gay or lesbian?	73 (2.3%)	28 (3.3%)	379 (60%)	3 > 1, 2
Peer Victimization—How often the following happened at school in the last 30 days					
PV1.	Other students picked on me	63 (2.0%)	671 (78%)	389 (62%)	2 > 1, 3; 3 > 1
PV2.	Other students called me names	122 (3.9%)	708 (83%)	454 (73%)	2 > 1, 3; 3 > 1
PV3.	I got hit or pushed by others	52 (1.7%)	264 (31%)	259 (41%)	2 > 1; 3 > 1, 2
PV4.	Other students made fun of me	60 (1.9%)	678 (80%)	418 (67%)	2 > 1, 3; 3 > 1

^a^ Class 1 (67%)—Low across all outcomes; ^b^ Class 2 (18%)—Moderate sexual harrassment, high peer vict., and moderate to low homophobic vict.; ^c^ Class 3 (14%)—Highest risk across most outcomes; ^d^ All class comparisons are significant at *p* < 0.001; significance tests were adjusted for multiple pairwise comparisons using the Tukey correction.

**Table 6:** Percentage of Participants Classified in each Class According to Gender, Sexual Orientation, Race/Ethnicity, and Prevalence of the Protective Factors.

	Class 1 ^a^ *n* = 3249	Class 2 ^b^ *n* = 868	Class 3 ^c^ *n* = 661	Sig. Comparisons between Classes ^d^
Gender *				
Male	50%	43%	55%	1, 3 > 2
Female	48%	55%	38%	1, 2 > 3; 2 > 1
Other Gender	1%	1%	4%	3 > 1, 2
Transgender	1%	1%	3%	3 > 1, 2
Sexual Orientation				
Gay or Lesbian	2%	1%	5%	3 > 1, 2
Bisexual	5%	7%	17%	3 > 1, 2; 2 > 1
Questioning	3%	2%	4%	
Other Sexual Orientation	3%	3%	7%	3 > 1, 2
Race/Ethnicity				
Black or African American	2%	2%	2%	
Hispanic	49%	42%	37%	1 > 2, 3
White	35%	41%	42%	2, 3 > 1
Multiracial	11%	12%	14%	3 > 1
Other Race/Ethnicity	4%	4%	4%	
Protective Factors				
Medical Access	85%	74%	72%	1 > 2, 3
Counseling Access	80%	69%	64%	1 > 2, 3
Trusted Adults	85%	78%	73%	1 > 2, 3
Family Support	89%	82%	75%	1 > 2, 3; 2 > 3
Peer Support	93%	87%	83%	1 > 2, 3
Spirituality	75%	63%	53%	1 > 2, 3

Note: * Percentages indicate the proportion of participants in each class endorsing the item. ^a^ Class 1 (67%)—Low across all outcomes. ^b^ Class 2 (18%)—Moderate sexual harassment, high peer vict., and moderate to low homophobic vict. ^c^ Class 3 (14%)—Highest risk across most outcomes; ^d^ All class comparisons are significant at *p* < 0.05; significance tests were adjusted for multiple pairwise comparisons using the Bonferroni correction.

**Table 7:** Latent Class Multinomial Logistic Regression of the Likelihood of Class Membership with Class 1 as the Reference Class.

	Class 2 vs. Class 1			Class 3 vs. Class 1		
N = 4318	O.R.	S.E.	*p*	O.R.	S.E.	*p*
Protective Factors						
Medical Access	0.67 ***	0.09	0.00	0.77	0.12	0.06
Counseling Access	0.72 ***	0.09	0.00	0.70 ***	0.10	0.00
Trusted Adults	10.03	0.15	0.86	10.10	0.18	0.59
Family Support	0.84	0.13	0.22	0.64 ***	0.11	0.00
Peer Support	0.73	0.14	0.05	0.54 ***	0.10	0.00
Spirituality	0.73 ***	0.08	0.00	0.66 ***	0.08	0.00
Sexual Orientation						
Bisexual	10.08	0.22	0.72	60.01 ***	10.03	0.00
Gay/Lesbian	0.61	0.29	0.17	50.37 ***	10.47	0.00
Questioning	0.59 *	0.19	0.03	10.67	0.49	0.17
Other Sexual Orientation	0.98	0.28	0.94	30.59 ***	0.81	0.00
Age (years)	0.88 **	0.04	0.01	0.83 ***	0.05	0.00
Gender						
Female	10.36 **	0.13	0.01	0.46 ***	0.06	0.00
Other Gender	10.51	0.73	0.49	10.49	0.62	0.42
Race/Ethnicity						
Black or African American	0.92	0.35	0.83	0.74	0.34	0.44
Hispanic	0.71 ***	0.07	0.00	0.65 ***	0.08	0.00
Multiracial	10.02	0.15	0.88	0.97	0.16	0.86

Note: Bold indicates significant associations, *** *p* < 0.001, ** *p* < 0.01, * *p* < 0.05, O.R. = odds ratio, S.E. = standard error.

## Data Availability

Data for this study is not publicly available.
